# Hepatic Epithelioid Angiomyolipoma: Case Series

**DOI:** 10.4021/gr273w

**Published:** 2010-11-20

**Authors:** Hetal Talati, Jasim Radhi, Snezana Popovich, Michael Marcaccio

**Affiliations:** a Department of Pathology and Molecular Medicine, McMaster University, 1200 Main Street West, Hamilton, Ontario, L8N 3V7, Canada; b Department of Surgery, McMaster University, 1200 Main Street West, Hamilton, Ontario, L8N 3V7, Canada

**Keywords:** Liver, Epithelioid angiomyolipoma, Adenoma, Hepatocellular carcinoma

## Abstract

Hepatic epithelioid angiomyolipoma (AML) is a rare, benign, mesenchymal neoplasm found in both males and females, and most commonly encountered in adult females. These lesions are difficult to diagnose by imaging, especially when fatty component is scant or absent. Histomorphologically, they resemble hepatocellular carcinoma. The tumor cells are strongly positive for homatropine methylbromide-45 (HMB-45) and smooth muscle actin by immunohistochemistry, which are the key markers for accurate pathological diagnosis. Hepatic AML should be considered in the differential diagnosis of a well circumscribed hepatic mass, even in the absence of an adipose tissue component.

## Introduction

Angiomyolipoma (AML) is an uncommon benign mesenchymal tumor, found most often in the kidneys and has been associated with tuberous sclerosis [1]. Hepatic AML lesions are composed of varying amounts of smooth muscle cells, adipose tissue and vessels. The smooth muscle cell component is the most specific to the diagnosis of these lesions. These cells can have varying morphologies and express positive immunostaining for homatropine methylbromide-45 (HMB45), but are negative for hepatocyte paraffin 1(HepPar1) and S100 protein [2]. The definitive diagnostic study remains the histologic examination of the surgically resected lesion or biopsy coupled with immunohistochemical stains. The differential diagnosis includes hepatocellular carcinoma, hepatic adenoma, leiomyoma, hepatoblastoma, melanoma, and gastrointestinal stromal tumor. The behaviors of these tumours are generally benign, but exceptional instances of malignancy in AML and metastasis have been reported [3].

## Case Report

Three cases were identified from the review of our institutional database for mesenchymal liver tumors in the last 15 years.

**Figure 1:**
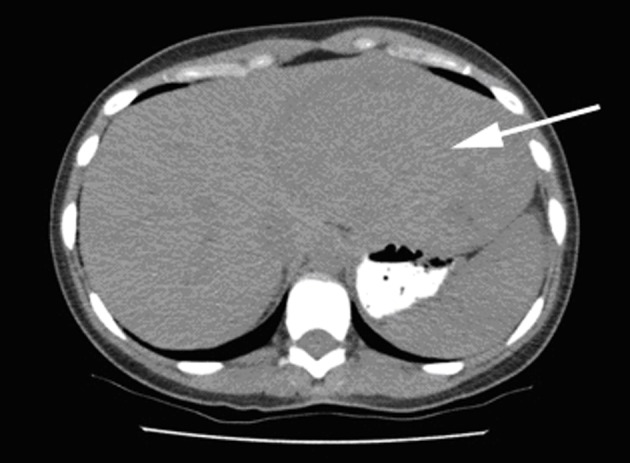
Liver lesion: CT image showing large hypodense lesion in the left liver lobe (arrow).

All three Patients in this series were females aged 38, 49, and 39 years presented with enlarging abdominal mass or masses. The most common symptoms were abdominal pain and discomfort. Case 3 presented with spontaneous hemorrhage into the lesion presented at one month postpartum. Laboratory investigations including liver function test were within normal limits in all three cases. CT scan imaging showed hypodense lesions (Fig. 1). Prior to surgery, diagnosis of hepatic AML was made following an ultrasound guided biopsy in one of the cases. Lipomatous element was absent in this biopsy specimen. These patients underwent segmental resections of liver. Intraoperative complication of bleeding was encountered in case 1, requiring 9 units of packed red blood cells and 7 units of fresh frozen plasma. Postoperative course was uneventful in all three cases.

**Figure 2:**
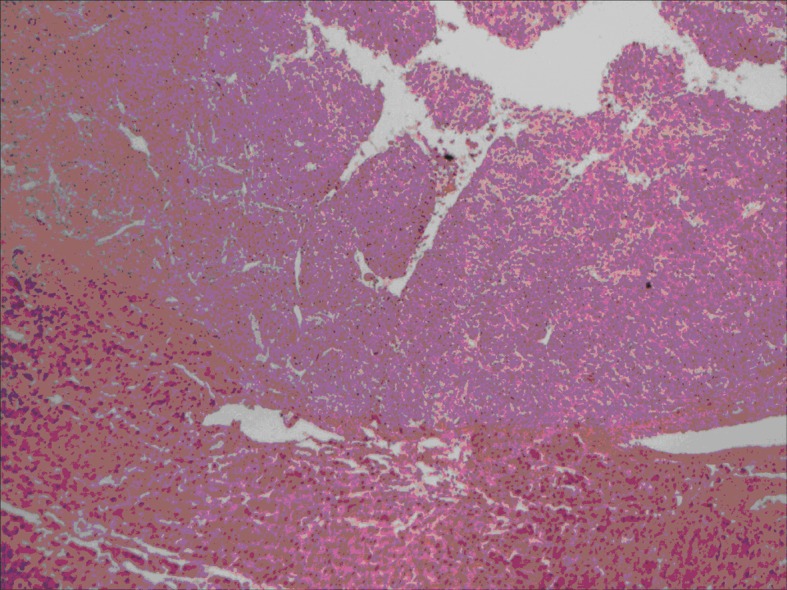
Section showing well circumscribed lesion (left) with surrounding normal liver tissue.

**Figure 3:**
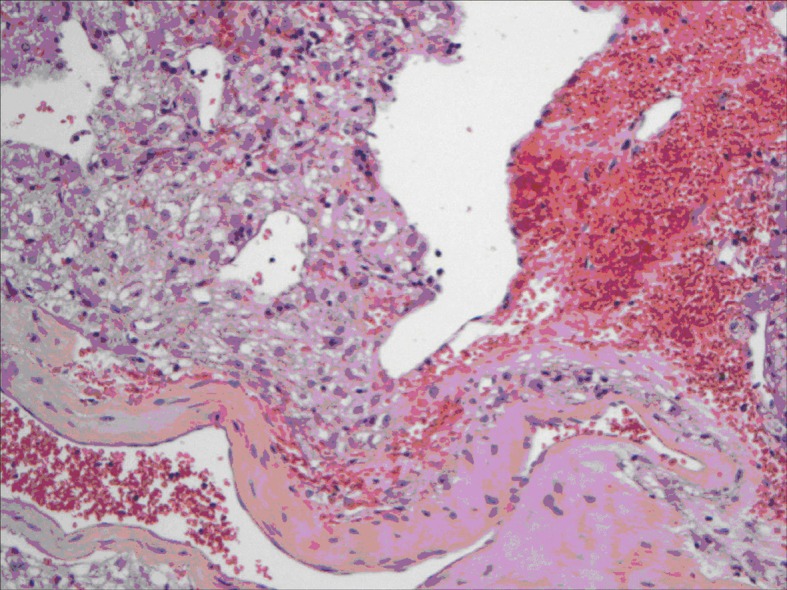
Epithelioid angiomyolipoma: tumor cells showing ample granular cytoplasm resembling hepatocyte with large vessel and hemorrhage.

**Figure 4:**
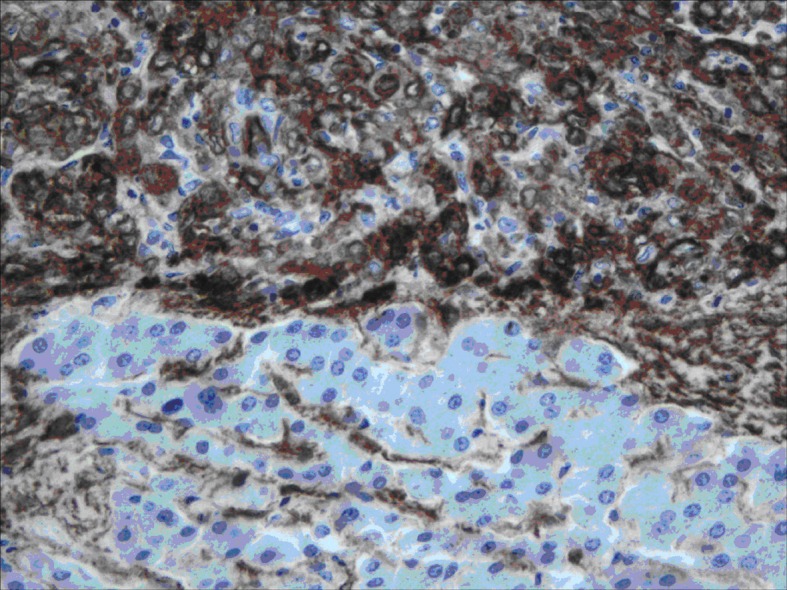
Immunohistochemistry staining: positive staining of the tumor cells for HMB-45.

All lesions were solitary, well circumscribed with soft yellow to tan color cut surface. Focal hemorrhage was evident in the large tumor. There was no necrosis noted, and the surrounding hepatic parenchyma appeared unremarkable. Light microscopy examination of sections from these lesions showed diverse cellular elements that are surrounded by normal looking hepatic parenchyma (Fig. 2). A component of mature fatty tissue was observed in resection specimen of two cases. The tumor cells had abundant, eosinophilic or clear cytoplasm with round to oval nuclei showing dispersed chromatin. Some cells did show characteristic “spider web” morphology. Thick walled blood vessels with large diameter were found within the tumor (Fig. 3). Immunohistochemical stains show strong positivity for HMB-45 (Fig. 4) and smooth muscle actin in all cases and stains for S-100, HepPar-1, and Desmin were negative. Electron microscopy of the lesions demonstrated premelanosome-like structures in the epithelioid smooth muscle cells.

The final diagnosis in all these cases was hepatic angiomyolipomas of epithelioid type. The follow-up of these cases exhibited a benign course with no sign of recurrence after a minimum following period of 36 months.

## Discussion

AML is a mixed mesenchymal tumor that usually occurs in the kidney [1]. Two types are described: isolated angiomylipoma and angiomyliopma that is associated with tuberous sclerosis. The lesion is a rare hepatic neoplasm, first described by Ishak in 1976 [2, 3]. Rare occurrences have also been reported in many sites including the uterus, retroperitoneum, mediastinum, renal capsule, the hard palate, the nasopharyngeal cavity, the buccal mucosa, fallopian tube, vagina, penis, skin, abdominal wall, stomach, and spinal cord [4, 5, 6] The tumor is characterized by a triad of tortuous, thick-walled blood vessels, smooth muscle cells, and adipose tissue in varying proportions. Traditionally, the lipomatous tumors of the liver are designated according to the elements present: lipoma, hibernoma, angiolipoma, myelolipoma, angiomyelolipoma, angiomyolipoma, and angiomyomyelolipoma [2]. Monomorphic variants, composed almost exclusively or predominantly of smooth muscle cells and with less well pronounced blood vessels, have been described. These lesions can mimic leiomyoma or other benign or malignant smooth muscle neoplasms [5]. This heterogeneity makes the preoperative diagnosis difficult by imaging, needle biopsy, and other techniques. Although, the imaging features can be highly characteristic, the preoperative diagnosis was mistaken in more than half of the cases in the most reported series [6]. On ultrasound examination, most show heterogenous hyperechoic lesion indistinguishable from hemangioma [7, 8]. Contrast enhanced spiral CT shows marked enhancement of the soft tissue components in the arterial phase and portal venous phase. MRI is the most specific imaging entity for the detection of lipomatous component, but AML can show marked variation in the amount of adipose tissue present. Although imaging tools may help in the diagnosis, the definitive diagnostic tool remains the histologic examination of the surgically resected specimen together with the application of the appropriate immunohistochemical stains or markers. The pathological differential diagnosis of these lesions includes hepatocellular carcinoma, hepatic adenoma, leiomyoma, hepatoblastoma, melanoma, and gastrointestinal stromal tumor. The smooth muscle cell component is the most specific to the diagnosis. The cells can exhibit in epithelioid, spindle, or intermediate forms. In addition to the morphological variation of the cellular components, hepatic AML can also assume various patterns, such as trabecular, solid, or inflammatory [2]. The epithelioid appearance of the tumor cells with trabecular growth pattern can be confused with a diagnosis of hepatocellular carcinoma, especially pertaining to small biopsies [9]. However, attention to the cellular details and immunohistochemical stains aid in arriving at the correct diagnosis. The epithelioid cells in AML possess clear cytoplasm with “spider web” morphology and are positive for HMB-45 and SMA, but are negative for Hep Par1 and S100 protein. Following the accurate diagnosis hepatic angiomyolipoma, it can be treated conservatively or resected surgically. Almost all reported cases in the literature show no propensity for metastasis or malignant potential. Rare cases have been reported with omental and lung metastasis [10, 11]. Multiple hepatic angiomyolipomas are often found in patients with tuberous sclerosis and particularly in patients with bilateral diffuse renal angiomyolipomas. Multiple hepatic angiomyolipomas are stigmata of tuberous sclerosis, and their finding should not be surprising or provoke unnecessary biopsy in a patient with known tuberous sclerosis. The finding of multiple hepatic angiomyolipomas in a patient not diagnosed with tuberous sclerosis is reason to evaluate this patient for further evidence of tuberous sclerosis [12].

In conclusion, hepatic epithelioid AML are rare mesenchymal tumours, which require careful clinical pathology correlation in order to reach a definite diagnosis. AML should be considered in the differential diagnosis of a well circumscribed hepatic mass lesion even in the absence of adipose tissue component.
